# Psychedelics and neonihilism: connectedness in a meaningless world

**DOI:** 10.3389/fpsyg.2023.1125780

**Published:** 2023-08-09

**Authors:** Patric Plesa, Rotem Petranker

**Affiliations:** ^1^Department of Psychology, Slippery Rock University of Pennsylvania, Slippery Rock, PA, United States; ^2^Department of Psychology, McMaster University, Hamilton, ON, Canada

**Keywords:** psychedelics, neonihilism, psychotherapy, meaning, connectedness

## Abstract

The resurgence of psychedelic research explicitly targets treating mental health conditions largely through psychedelics-assisted psychotherapy. Current theories about mechanisms of change in psychedelics-assisted psychotherapy focus on mystical experiences as the main driver of symptom improvement. During these mystical experiences, participants report an enhanced sense of salience, connectedness, and meaning. Simultaneously, a growing psychedelic culture is also cultivating the use of psychedelics as medicine for relieving symptoms of anxiety and depression and promoting cognitive functions. We argue that an integral part of the excitement around the resurgence in psychedelics is in response to a meaning and alienation crisis that correlates with rising rates of anxiety and depression. Framing the absence of meaning as neonihilism, a contemporary correlate to the 19^th^-century phenomenon with unique features present in a neoliberal cultural context, we explore whether psychedelics combined with group therapy can provide answers to modern experiences of meaninglessness. Based on this exploration, we suggest concrete next steps both in the theory and practice of psychedelic psychotherapy toward what we are calling neonihilistic psychedelic group psychotherapy.

## Introduction

The Global North is going through a mental health crisis of a magnitude that has not been measured since scientists started measuring mental health (Rudd and Beidas, 2020). We speculate that clinicians may be increasingly overdiagnosing their clients for various reasons, including the need to bill insurance, a human desire to categorize the world, and expectations from professional associations and insurers to have an “indication” to target (Moynihan et al., 2012; Rogers and Mintzker, 2016; Thombs et al., 2019). Other reasons for the rise in diagnoses include increased public awareness of the importance of mental health, increased access, and the ever-growing number of diagnoses in the Diagnostic and Statistical Manual of Mental Disorders (Kawa and Giordano, 2012). An account that does not conflict with the possibility of overdiagnosis is that there is another factor affecting the worsening mental health trend we are observing: a meaning crisis.

**Figure 1:**
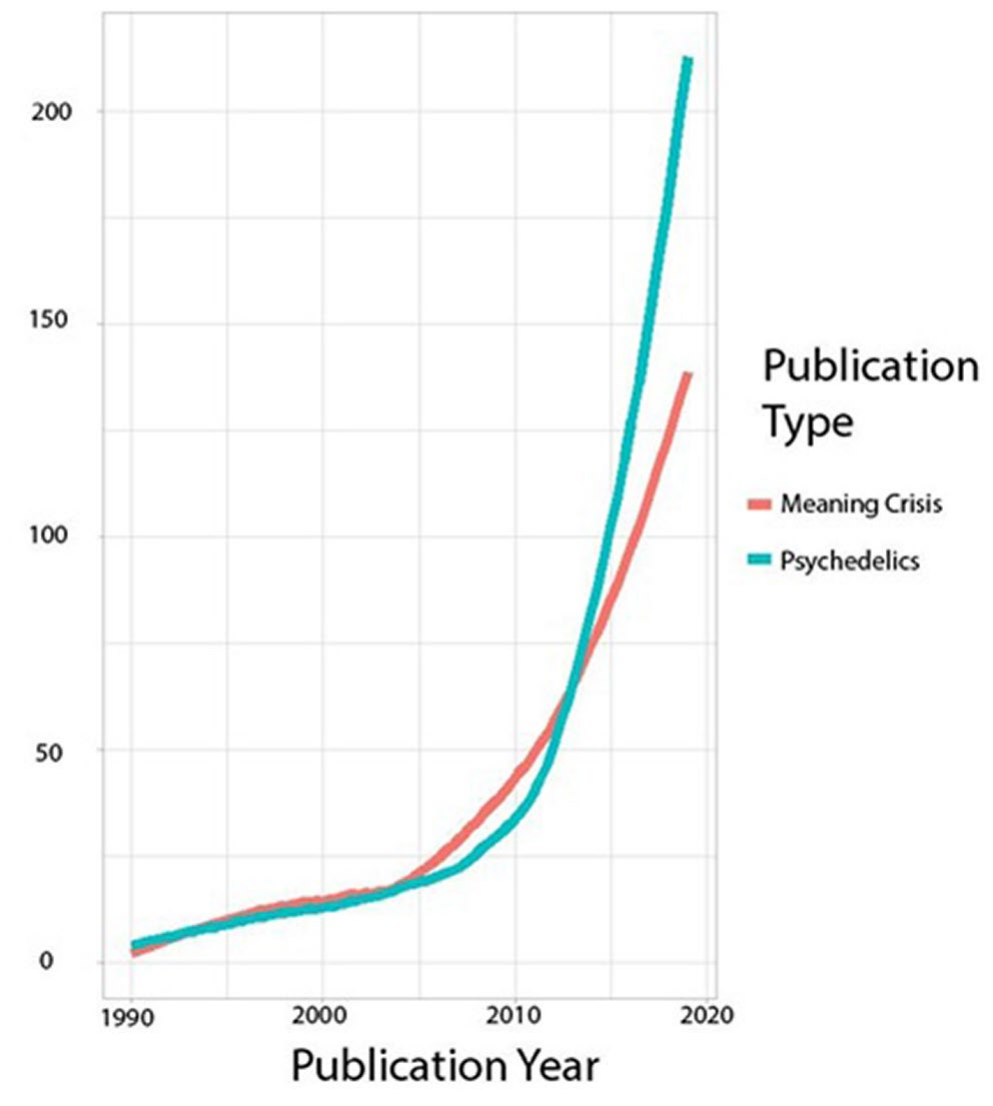
Comparison of publications including the words “Meaning Crisis” and “Psychedelics” over time. Number of publications with the keywords “Meaning Crisis” was divided by 10 to more clearly show the similarity in trends.

Publications focusing on a “meaning crisis” have increased exponentially over the last 20 years, which tells us that concerns over the loss of a sense of meaning is—at least academically—popular (see Figure 1, below). We also note that although correlation does not imply causation, publication trends regarding the crises of mental health and meaning in the last few decades mirror each other closely (Petranker et al., 2022). It is also possible that meaninglessness has become trendy: popular culture has identified this phenomenon as fashionable nihilism (Abumrad, 2014). Part of the popularity of nihilistic sentiments is a response to pervasive experiences of meaninglessness with a nonchalant attitude of rueful acceptance. The so-called meaning crisis and fashionable nihilism offer insight into a growing awareness of meaninglessness, which we argue is also related to the mental health crisis (Rudd and Beidas, 2020).

Shortly after the observed increase in the meaning crisis-related publications, an exponential increase in psychedelic publications emerged (Petranker et al., 2020). This resurging wave of psychedelic research focuses almost entirely on therapeutic interventions meant to combat mental health symptoms, portraying psychedelics as a much-awaited novel tool for addressing mental health concerns (Noorani, 2020). The mechanism through which psychedelics work remains unknown, although some theoretical and therapeutic notions have been posited (Hartogsohn, 2018; Carhart-Harris and Friston, 2019; Watts and Luoma, 2020). The consensus, at least at the moment, is that the subjective experience of undergoing a meaning-enhancing mystical experience, which includes a sense of oneness or connectedness to nature, divinity, or others, is conducive to improved mental health (Ko et al., 2022). Subsequently, much of the cultural excitement around psychedelics and support for ongoing research into their therapeutic effects rests on subjective and normative experiences of the enhanced subjective sense of meaning associated with psychedelic use (Hartogsohn, 2018).

Part of the cultural lore around psychedelics is a countermeasure to a subjective sense of meaninglessness with a deliberate search for meaning via psychedelic experiences. We identify two problems at this intersection: first, conceptions of meaning are often incoherent, and the subjective experiences of connectedness and salience during psychedelic trips may not effectively respond to either the mental health crisis or the meaning crisis in isolation. In the first instance, meaning is intrinsically subjective and difficult to operationalize consistently, and additional epistemological inquiry is needed to understand what meaning is in the context of psychedelics. In the second instance, psychedelics research is constrained in the clinical domain by the medicalization of psychedelic substances, offering the potential for mere symptom relief for what may be a mental health crisis in response to systemic conditions. In addition, this approach may ultimately cause more harm than good. For example, symptom-focused medicalization may not be tuned to the potential negative long-term effects of psychedelics given the lack of research on long-term effects (Schlag et al., 2022). Medicalization may run contrary to the mechanism behind the putative effects of psychedelics by focusing on symptom relief of what may be existential crises reframed as mental health (Noorani, 2020). Ultimately, medicalization can lead to the exclusion of existing knowledge and best practices drawn from indigenous and underground communities (Williams et al., 2021). Alternatively, popular psychedelic culture is often constrained by unsystematic experimentation, lack of oversight, mystification, or spiritual and cultural fetishization (Plesa and Petranker, 2022). The relationship between meaning and psychedelics is everywhere implied; however, we do not currently have any rigorous analyses of this relationship, perhaps due to the lack of a precise definition of meaning.

Our aim in this paper is first to begin from a place of negation, in describing meaninglessness rather than meaning, given that the former can better inform us what is absent from the broad landscape of meaning. With a working definition of meaninglessness as a series of missing pieces, we can better identify whether the salience and connectedness induced by psychedelic experiences can locate those missing pieces to alleviate feelings of meaninglessness. Framing meaninglessness historically in a lineage of nihilism will help us understand what conditions contribute to the absence of meaning and what remedies have been attempted before. With this historical lineage, it is also possible to frame our contemporary meaning crisis as a form of neonihilism, which we will define shortly, along with the competing notion of fashionable nihilism. Then we can consider what possibilities psychedelics pose for countering feelings of meaninglessness.

Second, we argue that part of the causes of the meaning crisis are systemic issues that fall on intersectional lines around racial, gender, sex, disabilities, and class inequities. There is sufficient literature to support the argument that systemic issues contribute significantly to the growing mental health crisis (Stewart et al., 2001; Pickett and Wilkinson, 2015; Taylor, 2016; Miu and Moore, 2021), and the meaning crisis (Rankin et al., 2009; Schnell, 2009). If the meaning crisis is caused by systemic issues, and psychedelics are being framed as a tool or treatment toward finding subjective meaning, then the enterprise is hopeless, because subjective meaning cannot fix systemic issues. We are helpless to change systemic issues with psychedelics, so we treat the individualized symptoms, even though meaninglessness may be experienced due to increased individualization, responsibilization, competition, and self-governance in neoliberal society (Beck and Beck-Gernsheim, 2002; Teo, 2018a).

Given that psychedelics are reported to increase feelings of connectedness (Carhart-Harris et al., 2018; Petranker et al., 2022), we can resolve to approach psychedelics from the point of connectedness as an opportunity to discover meanings collectively. We propose that the sense of subjective meaning elicited by psychedelics emerges from this sense of connectedness, which presents two therapeutic options: building collective meanings that have the potential to provoke resistance to the systemic issues causing meaninglessness, or short-term symptom-focused solutions that obscure the need for systemic change. Connectedness and collective meaning are worth exploring, even if resistance to systemic issues is only one, among many other possibilities.

Below we propose that two forms of psychedelics mirror two forms of modern nihilism, each attempting to tackle the contemporary sense of meaninglessness related to poor mental health. The direct effect of psychedelics can be used to alleviate the symptoms of the meaning-mental-health crisis, but they can also be used as a community-building tool to restore a sense of connectedness and salience to the world. Similarly, one form of contemporary nihilism produces a coping mechanism for meaninglessness, while the other suggests the possibility for confronting meaninglessness by means of connectedness.

## Neonihilism, meaninglessness, and connectedness

Neoliberalism began in the 1980s as economic policies of *laissez-faire* free-market capitalism, which transitioned into neoliberal cultural norms of individualization, responsibilization, competition, and self-governance (see Harvey, 2005; Brown, 2015). Neoliberal cultural logic internalizes systemic inequities and related problems of climate change, economic instability, and mental health into the self as personal responsibilities, inadequacies, and moral failures (Illouz, 2008; Fullagar, 2009). Neoliberalism as a set of cultural norms takes individuals out of the social world to seek change within the self, ushering a new nihilism that makes social change seem impossible (Teo, 2018a,b). Meaninglessness and mental health crises then appear as individualized and responsibilized internal conditions that one must address through self-governance and self-improvement strategies while competing for resources in a flexible market economy under the illusion of scarcity (Fisher, 2009; Figueroa, 2019). These conditions are exacerbated through global internet access in the information age when we are oversaturated with content and made hyperaware of ongoing social inequities, crises, and our seeming impotence to create change (see Scott et al., 2017; Corvalan et al., 2022).

Neonihilism is the confrontation of meaninglessness experienced under the contemporary material conditions of the 21st century, including social, economic, and political inequities. The arc of neonihilism terminates with finding irony–rather than satire– through a complete confrontation of the insurmountable barriers to a good life presented by current neoliberal agendas and market forces. Irony is a contradiction between a surface meaning and an underlying meaning (Fowler and Fowler, 1906). The surface meaning in neonihilism presents our social world and its problems as beyond our control, while the underlying meaning points to our individual responsibility for our condition within our social world, despite the contradiction. The sense of irony in the futility of tackling systemic problems individually is shared among others who confront the same meaninglessness and provides a point of connectedness that can be mobilized toward community building. However, when one becomes overwhelmed with feelings of meaninglessness and alienation, one may turn to fashionable nihilism. Fashionable nihilism contains the appearance of nihilism but focuses on producing satire out of meaninglessness as a coping mechanism, and maintains the status quo. In order to fully understand the relevance and underpinnings of neonihilism, we propose that a brief historical overview of the lineage of nihilism may be helpful.

19th-century nihilism was a form of meaninglessness due to lack of faith in God and the experience of poverty under industrialism (Hingley, 1969). Philosophers recommended a return to faith, not as a form of relief, but as a way of transforming a “suffering from” to a “suffering for,” thus offering us purpose (Jacobi, 2009; Kierkegaard, 2010). Nietzsche (1969) reoriented our “suffering for” away from the supernatural and toward a confrontation with ourselves as a form of empowerment toward creating our own meanings and purpose in life. 20th-century nihilism begins as a resistance to industrialism and becomes an existential phenomenon that considers life inherently meaningless (Frankl, 1984). Sartre (1992) and Camus (1955) suggest that we create meaning from our confrontation with meaninglessness. 21st-century nihilism is a response to a neoliberal status quo that places responsibility on the individual to create meaning in their labor and the self in lieu of the possibility for systemic changes (see Harvey, 2005).

Within this brief historical overview, we see the transition of nihilism as a social phenomenon combating spiritual, political, and material conditions, to a progressively internalized sense of meaninglessness driven by a sense of hopelessness to change the external conditions paradoxically responsible for our sense of meaninglessness. Within the new neoliberal form of nihilism, exists also the absurdity that the external conditions we are hopeless to change are internalized as personal moral failings (Fullagar, 2009), which further elicit feelings of meaninglessness. The recognition of this internalization is understood as irony, which is a by-product of our confrontation with neoliberal meaninglessness, and constitutes what we mean by neonihilism. However, the possibility to commodify meaninglessness in neoliberalism transforms that irony into satire, which acts as a coping mechanism to temporarily relieve the symptoms of meaninglessness, and constitutes what we mean by fashionable nihilism.

### Neonihilism and fashionable nihilism

Neonihilism can be understood from Nietzsche’s (1967) perspective on Greek tragedy, which elicits an uplifting element, or sense of pleasure, in confronting suffering. In the same way, neonihilism as a confrontation with meaninglessness elicits a sense of irony that is pleasurable, insofar as it is satisfying to understand the source of meaninglessness. This understanding via irony provides certainty as a sense of closure (Webster and Kruglanski, 1994), which ceases ambiguity and also the burden of hope. Within neonihilism, what is ironic is that one’s sense of personal meaninglessness is derived from hopeless external conditions, and despite this recognition, the meaninglessness persists and the available avenues for treatment are individualized, confirming the absurd notion once more that the problem is within the self. It is ironic to confront meaninglessness with hopelessness.

Fashionable nihilism shares with tragicomedy the uplifting element of comedy as a satire of the tragic to relieve the effects of suffering rather than confront them. Aristotle (2006) complained that tragicomedy was invented because the masses could not stomach the superior pleasure of tragedy and needed relief through the inferior pleasure of comedy. The satirical element in fashionable nihilism is the caricaturing of meaninglessness as something readily recognizable but diffused of its power to provoke deeper understanding or action. Furthermore, within neoliberalism, fashionable nihilism is a commodification of the diffused sentiment of meaninglessness as satire that profits from the marketability of its grim nonconformity. It is a posturing of the darkness of nihilism without understanding its existential effects (Abumrad, 2014), creating a transient sense of camaraderie without developing community. Carr (1992) describes this type of “cheerful nihilism” as an acceptance of meaninglessness without critical thinking. In this way, satire acts as a coping mechanism for the effects of meaninglessness in fashionable nihilism.

What this tells us is that the power to confront meaninglessness as a form of resistance to nihilism is obscured by the very system responsible for what we argue is the cause of meaninglessness, neoliberal capitalism. Progress toward meaning within neoliberalism becomes incoherent as an internalized problem. We cannot readily decipher if and when the threshold between the ironic confrontation and the satirical encounter has been crossed. Thus, we propose that externalizing the process by relying on the connectedness in the communal recognition of irony in neonihilism, may prove to be a more viable way to tackle the problem. What neonihilism invites, via the connectedness in irony, is a chance to create or discover meaning as a collective, rather than an individual experience. Connectedness becomes the avenue for collective meanings toward resistance to systemic oppression. It is to find resistance to the individualizing and responsibilizing power-relations of neoliberalism in collective modes of meaning-making via increased connectedness and salience. We argue that similarly, enhanced connectedness and salience are some of the main mechanisms through which psychedelics operate to increase a subjective sense of meaning and, through it, wellbeing.

## Meaning, connectedness, and salience in psychedelics

The study of psychedelics, especially in the current wave, has focused largely on their biochemical mechanisms (e.g., Nichols, 2016, 2020; De Gregorio et al., 2018), although recent work has focused on extrapharmacological factors as well (e.g., Hartogsohn and Petranker, 2022), and publications on the societal factors are also mounting (e.g., Devenot et al., 2022; Plesa and Petranker, 2022). Psychedelics users report increased subjective feelings of meaning following their experience, which has been suggested as a required mediator for improved mental health (Yaden and Griffiths, 2021). We posit that this sense of meaning emerges from direct changes to senses of connectedness and salience rather than existing as an independent phenomenon. There are at least two reasons to probe the determinants of psychedelics-occasioned meaning further: a better understanding of the psychedelic mechanism of action will lead to both better scholarship and better therapies. We propose that although set and setting–the mental and physical space in which psychedelics are used–has received much attention in the literature, the cultural-social context, or matrix, is required to characterize and optimize the psychedelic experience, building on the work of Eisner (1997). We employ a biopsychosocial model to track the potential mechanisms of action for psychedelics through the lens of neonihilism below.

### The neurobiological aspects of psychedelics and meaning

Before discussing the biological perspective, it is important to note that the number of imaging studies on the effects of psychedelics is small, but since certain trends arise from these samples we are inferring from them with an approach of intellectual modesty. One of the most consistent findings in the neuroscience of psychedelics suggests that the Default Mode Network (DMN), which is related to one’s sense of self, is disrupted, leading to increased connectivity between different brain areas which are not normally connected (Gattuso et al., 2022). It may also be related to feelings of “oceanic boundlessness” and “experiences of unity” often described by psychedelics users (Studerus et al., 2010). These have also been understood as a newfound connectedness to oneself, one’s community, and one’s values, especially in the context of an increased sense of personal meaning (Petranker et al., 2022). An additional, equally plausible account suggests that the disruption to DMN activity leads to more bottom-up modes of processing, which may have a therapeutic effect (Aqil and Roseman, 2023). In bottom-up processing, systems reduce the weight of prior knowledge and instead focus on trends that emerge from the data available in the moment. Thus, one is able to rely less on previous working theories about one’s place in the world and more on the data the world presents. In addition to the literature discussing the DMN, however, there is growing literature examining another brain network whose activity may be modulated by the effects of psychedelics.

The salience network, which is involved in the detection and integration of stimuli, is also affected by psychedelics use. This network is crucial for attending to what is happening in the environment, helping us decide which environmental stimuli should “matter” to us. This network has been implicated in several psychopathologies including anxiety (Xiong et al., 2020) and post-traumatic stress disorder (Akiki et al., 2017). In addition, this network also modulates the abovementioned DMN. Recent research has shown that using psychedelics also modulates the behavior of this network (Pasquini et al., 2020; Madsen et al., 2021), which may shift one’s salience landscape, changing what is important in the world. While under the influence of psychedelics, one’s relationship to stimuli in the world shifts, which may be part of the therapeutic mechanism of change (Singleton et al., 2022). There is a rich literature describing the increased sense of “mattering” in the world during and after psychedelics use (for a fuller discussion see Hartogsohn, 2018), which has often been equated to meaning, but as we show here, there is a distinct brain network associated with these subjective reports.

The neurobiology of the psychedelic experience suggests that there is no “meaning” network that is affected by these substances. Instead, two main related networks are involved: the DMN which reflects our ability to sustain a consistent sense of self, and the salience network which helps determine what matters to us in the world. Although research on the importance of both of these aspects of psychedelic experience has been explored, to our knowledge, no work has suggested that the subjective sense of meaning is an emergent property of the synergy between increased connectedness and salience. While a scale of connectedness was recently developed as a means to explore the importance of this aspect of the psychedelic experience (Watts et al., 2022), and some have implicitly and moderately equated salience with meaning (Hartogsohn, 2018), we argue that meaning as experienced via psychedelics is directly produced by changes in salience and connectedness. We move on to the next level of analysis to examine whether a psychological account includes a more direct explication of the meaning induced by psychedelics that can be directly accessed.

### The psychological aspects of psychedelics and meaning

The study of meaning in psychedelics has paralleled the study of meaning in psychology overall, serving as a general guiding principle without a rigorous definition. For example, self-determination theory which posits that well-being is derived from competence, autonomy, and relatedness (Ryan and Deci, 2000), has been linked to a feeling that things in the world are meaningful or devoid of meaning (Vansteenkiste et al., 2018). Others have considered meaning as a dynamic process which can be separated into presence vs. a search for meaning (Steger et al., 2008) using the framework of Frankl (1984). However, this line of research does not deeply engage with a definition of meaning either, instead focusing on the practical implications of their definitions of meaning. The same goes for some of the best minds in psychedelic research, who consistently report that psychedelics enhance a subjective sense of meaning without an explicit theory of meaning (Carhart-Harris et al., 2016; Preller et al., 2017). Instead, the psychological investigations of the effects of psychedelics rely on a subjective sense of “personal relevance” (Preller et al., 2017) or specifically rely on the 5-Dimension Altered States of Consciousness scale, which directly asks about “altered meaning” and whether “things in the environment acquire a special meaning” (5D-ASC; Studerus et al., 2010, p. 9). While these descriptions sound akin to increased salience, a growing literature on the importance of subjective feelings of connectedness suggests that subjective feelings of belonging are ubiquitous following the use of psychedelics.

A variety of naturalistic studies found increases in self-reported connectedness related to the use of psychedelics (Forstmann et al., 2020; Weiss et al., 2021; Petranker et al., 2022), and connectedness is canonically related to well-being, especially in the context of psychedelics (Watts et al., 2017). The precise definition of connectedness and its relationship to meaning remains elusive, however, Forstmann et al. (2020) examined the “Inclusion of Self in Others” (Aron et al., 1991), in which participants select how much their sense of “self” overlaps with another. Petranker et al. (2020) used a qualitative analysis of respondents to a survey to construct a theory rather than impose one, thus relying on participants’ personal definition of “connectedness.” Weiss et al. (2021) found the same trend by using the Social Connectedness Scale (SCS; Lee and Robbins, 1995) which measures a perceived sense of belongingness and is focused on psychopathology (Lee and Robbins, 1995), and the Inclusion of Self in Others scale used by Forstmann et al. (2020). Even the new Watts Connectedness Scale (Watts et al., 2022), which is informed by extensive experience in the field and numerous reports, does not clearly connect connectedness to meaning. Based on this literature, we conclude that while there seems to be a relationship between connectedness, salience, meaning, and well-being, its specifics remain unknown and is not currently supported by placebo-controlled trials.

The literature about shifts in salience is more conclusive and appears to generally–but not always–suggest that these shifts lead to a sense of meaning and well-being, especially if drawn from resources outside of psychedelics. In the psychedelics literature, it is difficult to demarcate salience-specific changes from other brain and mind-related changes since the impact is holistic. Outside of psychedelia, bottom-up processing via a shift in salience is considered one of the main mechanisms through which mindfulness improves well-being in the case of trauma (Westerman et al., 2020) and anxiety (Treanor, 2011). These shifts in salience focus have also been argued to cause an increased sensed feeling of meaning in life (Chu and Mak, 2020). Similarly, near-death experiences may cause a sudden shift in one’s salience landscape, leading individuals to value empathy and spirituality more (Greyson, 2006). This comparison is particularly relevant as psychedelics such as DMT (dimethyltryptamine) arguably model near-death experiences both phenomenologically and in their impact (Timmermann et al., 2018). It is not merely a change in brain network activity, then: it appears that the change in attitudes and behaviors following a shift in one’s salience landscape creates a sense of felt meaning. Using a reductionist analysis of “brain only” and “mind only” has only been partially effective in disambiguating the relationship between connectedness, salience, and meaning. It may be that a larger societal prism is required to reconstruct these parts into a coherent narrative.

### The social aspects of psychedelics and meaning

The duo of set and setting, extant from the first wave of psychedelics research, were originally part of a trifecta: set, setting, and matrix. Eisner (1997) defines the matrix as “the environment from which the subject comes… and the environment to which a patient returns after successful therapy.” (p. 215). Under this definition, the social context, including our friends, family members, and work environment, as well as values, beliefs, and attitudes, all play into the impact of psychedelic use. In addition, our cultural values, mores, and norms shape this matrix and the ways in which psychedelics may affect us. We propose that the meaning crisis in our current neoliberal social matrix is related to feelings of alienation, and a lack of clarity regarding what matters in life. While these feelings are not new, their systemic and seemingly omnipotent determinants appear impregnable. Creating an optimal set and setting and then using a substance that may affect one’s sense of connectedness and salience before releasing them back to a matrix of a meaning crisis is a temporary solution at best. Furthermore, it perpetuates the myth that the only change one can make is internal and subjective, similar to the mindfulness literature, which has implicitly suggested that by working on oneself, one’s society may change (du Plessis and Just, 2022). As far as the twin meaning and mental health crises, however, it does not appear that our society is improving by practicing more mindfulness or using more psychedelics yet (see Figure 1). Instead, our current matrix of neoliberalism, with its cultural assumptions of individuality, self-sufficiency, and materialism, is flying in the face of the mechanisms underlying the putative effects of psychedelics by undermining possibilities for connectedness. As we argue above, the enhanced meaning experienced by psychedelics users, which leads to improved mental health, is predicated on a stronger sense of connection and a shift in one’s salience landscape, while the neoliberal matrix espouses the exact opposite.

If connectedness is indeed necessary for the meaning-induced improved mood observed in psychedelics users, then our contemporary neoliberal matrix requires acknowledgement and mitigation for optimal psychedelic psychotherapy. Most courses of psychedelic psychotherapy focus on preparation for the journey, the psychedelic experience itself, and the subsequent integration of the experience into one’s life (Bathje et al., 2022). However, this approach largely disregards the wider context of the therapy: even if one feels a greater sense of connection to their self, their community, or the world, they will still return to a neoliberal culture which enshrines individualization, responsibilization, competition, and self-governance (see Beck and Beck-Gernsheim, 2002; Teo, 2018a). This is particularly true of the medicalized pathway through which psychedelics are currently dispensed and the therapy methods that are almost exclusively focused on individual therapy not only in the therapy room, but also, outside. Clients arrive and leave individually and return to their communities with the impossible task of communicating an ineffable experience and its effects on them to their families and friends or, alternatively, processing the experience alone. Indeed, undergoing what is frequently referred to as a life-changing experience (Carhart-Harris, 2019), which is inexplicable to one’s social circle, may even serve to distance one from their community, as reported by mindfulness meditators (Anderson et al., 2019). The alienation matrix is likely detrimental to the action of psychedelics, but the way in which shifts in one’s salience landscape may be coopted by the neoliberal matrix is more pernicious.

When undergoing psychedelic psychotherapy, one’s salience landscape shifts, making one more likely to consider certain things as “important,” which fueled some of the initial enthusiasm about these substances for “mental manipulation” (Dupuis, 2021). This manipulation may be useful if the issues one wishes to tackle in therapy are endogenous, as clients can be helped to change how they process self-referential information (e.g., one’s excessive feelings of anxiety are taken in a wider perspective). However, consider the rise in climate-related anxiety (Taylor, 2020) as a case study of the potential detriment of using psychedelics to encourage responsibilization rather than collective action. A client comes in from the neoliberal matrix in which the climate is changing, full of anxiety about the upcoming years and decades. The client reports a sense of meaninglessness: there is no way to stop climate change, the negative outcomes are inevitable, and hopelessness about the future is mounting. One way of using psychedelics to help this client is to shift the salience of this ongoing issue from an external event that needs to be addressed by way of action into a subjective, internal event that the client must learn to accept because they cannot change it. Another way of using psychedelics to help this client is to shift the salience of the event from the personal to the collective, using a two-step process: first, by helping the client confront the incredible magnitude of the problem and the likely-insurmountable systemic changes that are required to solve it, and then by reminding them that the notion that they must change the world on their own is part of their cultural matrix and that climate change is, in fact, a collective action problem. Furthermore, we argue that this second method is best applied in group therapy settings, which promote collective meaning-making and action.

We suggest that in the case of psychedelics, the current focus on set and setting should expand to contain the matrix of meaninglessness and encourage an engagement with these feelings via neonihilism–a *confrontation* with meaninglessness. Without such considerations, psychedelic psychotherapy may only temporarily solve felt meaninglessness without addressing its underlying cause, which would parallel the *encounter* with meaninglessness we have described in fashionable nihilism as a coping mechanism.

## Neonihilistic psychedelic group psychotherapy

Nietzsche (1974) argued that within nihilism we can find the power to overcome it. Similarly, within neonihilism exists the point of resistance in the collective recognition of irony, which is a form of connectedness that points toward the possibility for collective meaning-making. Neonihilism is merely a descriptive phenomenon of a contemporary form of neoliberal nihilism with a by-product of ironic futility to make systemic changes. However, by negation, we have arrived at the missing piece that results in meaninglessness; what we argue is missing is a collective salience landscape together with a sense of solidarity with others in tackling systemic problems. Psychedelics may help by linking one’s salience landscape with a feeling of connectedness that can be explored communally toward solidarity in collective–rather than individual– meaning-making. In this way, psychedelics offer an avenue to challenge neonihilism if we shift away from individual therapy and toward connectedness as a collective experience.

Our proposition is that the unifying principle of connectedness is the underlying mechanism beneath all of the concepts introduced here. Its absence is core from our neonihilistic matrix, instrumental to our contemporary meaning crisis, causal to the mental health crisis, and can be explored by using psychedelics. Using neonihilism as the social matrix in a trifecta including set and setting for psychedelic experiences, we propose group-oriented therapeutic interventions as an appropriate setting for optimizing the therapeutic outcomes by exploring connectedness. The bounds of psychedelic therapy should allow therapists to skilfully alert clients as needed to the internal and external causes of their conditions. When appropriate, therapy should focus on the client’s internal world and experiences and learning to accept them while holding space, as is the current best practice (Thal et al., 2022). However, when appropriate, the therapist should be able to also hold space for the collective systemic issues–the matrix–that provoke experiences of meaninglessness and orient the client toward the possibility for collective meaning-making. This novel approach to psychedelic-assisted psychotherapy will yield better results as it utilizes the underlying mechanisms of psychedelic action, moving toward connectedness via the shared neonihilistic social matrix in group settings. The therapeutic orientation toward a collective confrontation of meaninglessness is meant to subvert the individualizing, responsibilizing, and self-governing neoliberal ethos, which we argue is the recurring obstacle to effective strategies to combat the meaning and mental health crises via psychedelics.

In addition to the modifications proposed in the therapy room, we also suggest two structural changes: a deeper consideration of the client’s matrix, and a focus on group rather than individual therapy. There is no gold standard for psychedelic psychotherapy, which we proposed elsewhere should be addressed by decriminalization and support from professional organizations (Plesa and Petranker, 2022). Here we posit that any such gold standard should include more than collecting demographic information and presenting concerns, such as, which systemic or social issues the client faces, what they feel most hopeless about, and what supports they have in their lives. The therapist should be mindful of where the client is coming from and to whence they return, in order to prepare them to reintegrate into their milieu. In addition, group therapy may help create such a milieu so that clients do not have to go back to the same matrix from which they came, and instead make new connections salient via the therapeutic process.

The current evidence base suggests that meaning is important, if not necessary, to get benefits from psychedelics, and it appears that meaning is an emergent property of connectedness and salience. Thus, a focus on connectedness and salience in the therapy room should be key. We propose that the former can be addressed by focusing the above mentioned neonihilistic approach in group therapy, which lacks randomized control trial evidence, however, psychedelic group settings are the norm in many indigenous (Sabina and Wasson, 1974) and underground communities (Gasser, 2022). Using psychedelics in a group setting would of course present major cost savings which are currently desperately needed (dos Santos et al., 2021), but we propose that group therapy should be the norm because it dovetails with the psychedelic mechanism of action. We hypothesize that psychedelic psychotherapy, done in a group setting, will have a synergistic effect since the connectedness-enhancing process will be magnified. We expect that the group will become a more salient unit of cohesion which clients will benefit from substantially. Group members can also attend follow-up group sessions, form consensual accountability relations, and organize to meet outside of therapy for ongoing support.

Our suggestion for neonihilistic psychedelic group psychotherapy is relatively novel but not without precedent, at least in the first-generation psychedelics research. Nevertheless, the attempts at group therapies involving psychedelics between the 1950s and 1970s did not employ rigorous or homogenous methodologies. A review of clinical psychedelic group therapies from 1900 to 2018 by Trope et al. (2019) concludes that “methodological shortcomings common to this era of psychedelic research include lack of proper control groups, lack of blinding procedures, inconsistent diagnoses and treatments applied across groups, outcome measures that were either unvalidated or absent, and poor or absent statistical analysis” (p. 12), which leaves the efficacy of such therapies indeterminate. Nevertheless, the authors argue that recent studies in individual psychedelic therapies using empirical qualitative data show that social connectedness is a fundamental mechanism to therapeutic change, adding that “a group component in psychedelic research protocols serves as a significant manipulation of both attitudinal set and environmental setting” (Trope et al., 2019, p. 13).

Since Trope et al. (2019) review, only a few studies have looked at psychedelic group psychotherapy (Gross, 2021; Gasser, 2022), the most notable of which is Anderson et al. (2020) clinical trial of psychedelic-assisted group psychotherapy with older long-term AIDS survivor (OTLAS) men, which has been heralded as trailblazing for using group therapy in combination with psychedelics to treat a marginalized community that faces an existential form of “demoralization—a sense of helplessness, hopelessness, and a loss of meaning in life” that is often comorbid with mental health conditions (Hendricks, 2020, p. 1). Although this is a pilot study, it indicates the feasibility of conducting psychedelic-assisted group psychotherapy and moreover the application of this therapeutic intervention to crises of meaning and mental health. Furthermore, group approaches to psychedelics-assisted psychotherapies have at least two major benefits: (1) Given that psychedelics-assisted therapies are time-intensive, group settings are more cost effective and scalable, (2) groups settings are optimal for meaning-making around such existential and ineffable experiences, which leads to more adaptive changes in behavior (Trope et al., 2019; Hendricks, 2020).

There is precedent for relating felt meaning to both connectedness (Carhart-Harris et al., 2018) and salience (Pasquini et al., 2020), the use of group therapy in tandem with psychedelics (Gasser, 2022), and the integration of a social matrix along with the established model of set and setting (Eisner, 1997). Our contribution here is twofold. First, we propose a new relationship between these constructs that disambiguates causal relationships and positions meaning as an emergent property of connectedness and salience. Second, in synthesizing these ideas together with our theoretical framework of neonihilism as the neoliberal social matrix, we create an explicit focus on sources of collective meaninglessness and the need for collective meaning-making as a viable solution, which can be therapeutically guided. Neonihilistic psychedelic group psychotherapy focuses on a collective confrontation of meaninglessness as a radical departure from individualizing therapeutic practices that further reinforce neoliberal forms of individualization, responsibilization, competition and self-governance. Our model for group therapy is ambitious, but proportional to our meaning and mental health problems, and requires some caveats and acknowledgement of limitations.

First, in guiding groups to confront meaninglessness toward collective meaning-making we are transparent about our theoretical assumptions underpinning neonihilism but we do not impose them on patients as a belief system. Our approach would focus on *how* to think about meaninglessness through connectedness with others rather than an individual phenomenon, not form a basis for *what* to think. Second, discussing solidarity and collective action as potential solutions to systemic problems only signifies the political relevance of our approach, not the method or expected outcome of our proposed group therapy. We recognize that psychedelics may not promote prosocial behaviors, but rather, amplify existing beliefs (Nour et al., 2017), which are likely to align with the neoliberal cultural values we argue are part of the problem of meaninglessness. Theorizing meaninglessness as a response to internalizing systemic issues in neoliberal capitalism is an important framework for understanding the value in collective approaches to meaning-making as an antithesis to continuing with individualizing approaches. As such, contextualizing group therapy to a shared social matrix shifts the focus to collective solutions for meaning and mental health crises to potentially arise.

## Conclusion

Psychedelic-assisted psychotherapy deals with connectedness and salience, from which meaning emerges. The existing literature points to set and setting as key factors in determining therapeutic outcomes, but excludes the social matrix that enmeshes psychedelics users, and to which they must return following therapy. This social matrix includes one’s social circles and culture, but also systemic problems, which we argue are internalized in neoliberalism and contribute to the meaning and mental health crises. We have theorized these crises as contributing to contemporary forms of nihilism, which is a sense of meaninglessness. A confrontation with meaninglessness produced by internalizing systemic problems results in the ironic conclusion that the individual cannot hope to make systemic changes in a highly individualized social world. At the same time, one feels responsible for changing and governing the self to cope with these problems. This confrontation with meaninglessness that produces irony is what we have called neonihilism; in contrast, the neoliberal commodification of nihilistic sentiments in media produces satire, which acts as a coping mechanism toward insurmountable systemic problems. The commodification of nihilism is what we have called fashionable nihilism, which is merely an encounter, rather than a confrontation, with meaninglessness.

Likewise, psychedelics-assisted psychotherapy can follow one of two paths. One path includes a brief encounter with meaninglessness that is then navigated back to intrapersonal coping, by focusing on the individual. Alternatively, as we have suggested, psychedelics-assisted psychotherapy can be a confrontation with meaninglessness that, rather than ending in irony, can explore connectedness together with others toward solidarity and collective meaning-making. We have looked at meaninglessness as an absence of meaning and suggested that what is missing is solidarity and communion with others that can be facilitated via the sense of connectedness indicated in neonihilism, and potentially responded to in the psychedelic experience. Exploring connectedness communally in therapeutic settings has the potential to create collective meanings about our shared social worlds.

## Author contributions

All authors listed have made a substantial, direct, and intellectual contribution to the work and approved it for publication.

## Conflict of interest

RP is a consultant for Naya Technologies Inc.

The remaining author declares that the research was conducted in the absence of any commercial or financial relationships that could be construed as a potential conflict of interest.

## Publisher’s note

All claims expressed in this article are solely those of the authors and do not necessarily represent those of their affiliated organizations, or those of the publisher, the editors and the reviewers. Any product that may be evaluated in this article, or claim that may be made by its manufacturer, is not guaranteed or endorsed by the publisher.
